# 

**Published:** 1887-03-12

**Authors:** 


PRICE ONE PENNY.

No. 24, Vol. I.

March 12th, 1887.

Registered at the General Post Office as a Newspaper.]

Per Annum, 6s. 6d., post free.
Monthly Parts, 6d.; post free, 7½d.
Ditto per Ann., 7s. 6d., post free.

### THE HOSPITAL

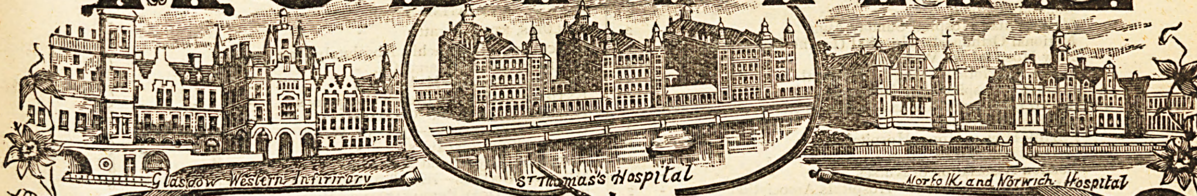

An Institution, Family, and Parochial Journal of

Hospitals, Asylums, & all Agencies for the Care of the Sick, Criticism & News.

